# Contrast-enhanced ultrasound of intrahepatic portal vein gas: Case report and review of literature

**DOI:** 10.1016/j.radcr.2021.04.056

**Published:** 2021-06-10

**Authors:** Tingting Sha, Tinghui Yin, Rongqin Zheng

**Affiliations:** Department of Medical Ultrasonics, Laboratory of Novel Optoacoustic (Ultrasonic) Imaging, The Third Affiliated Hospital of Sun Yat-Sen University, Guangzhou, China

**Keywords:** Portal vein gas, Contrast-enhanced ultrasound, Ultrasound, Liver blood flow, PVG, portal vein gas, CT, computed tomography, US, ultrasonography, CEUS, contrast-enhanced ultrasound, ER, emergency room, CECT, contrast-enhanced computed tomography

## Abstract

Portal vein gas is a rare imaging finding and a concomitant sign of abdominal disease. Here, we report a 64-year-old man with an emphasis on contrast-enhanced ultrasound for describing the findings for portal vein gas and evaluating liver blood perfusion. Ultrasonography is a favorable imaging modality for the rapid bedside evaluation and monitoring of portal vein gas in the emergency room.

## Introduction

Portal vein gas (PVG) is a rare condition that occurs when gas from the gastrointestinal tract or produced by bacteria enters portal venous circulation. The mortality rate of PVG varies depending on the etiology, including bowel ischemia, which requires emergency surgery and has a high mortality, and iatrogenic injury, which usually needs conservative treatment [1]. Early detection and active treatment can improve patient prognosis.

Most medical literature mentions computed tomography (CT) and conventional ultrasonography (US) (grayscale US, Doppler US and M-mode US) for describing the imaging manifestations of PVG. In most reported cases, CT is the primary imaging modality in the emergency room (ER) [2]. Bedside US, whichis portable, and convenient for use in the ER, is another sensitive imaging modality for detecting gas in the portal vein [3]. However, conventional CT or US without contrast enhancement is unable to capture much useful information, such as blood perfusion. Although contrast-enhanced CT (CECT) has been described in many articles, there are currently no reports of evaluating heterogeneous liver perfusion of PVG. To the best of our knowledge, there have been no reports of PVG evaluation by contrast-enhanced ultrasound (CEUS). Here, we report a 64-year-old man with PVG who was diagnosed by bedside CEUS. The aim of this report was to describe US (especially CEUS) imaging features of PVG and to outline the diagnostic value of CEUS.

**Fig. 1:**
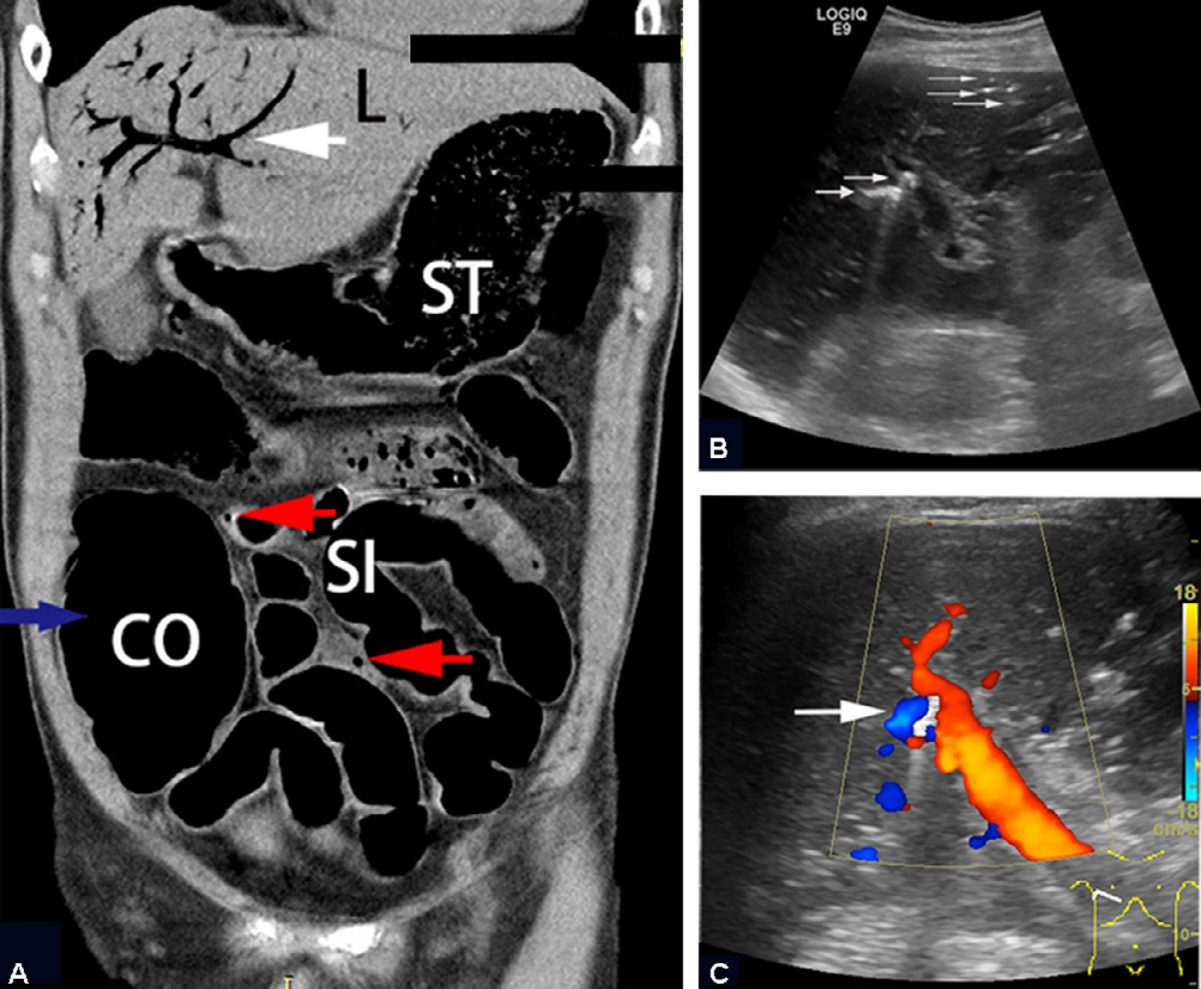
Imaging findings of PVG by CT and conventional US. (A) CT coronal view of liver with extensive hepatic PVG (white arrow), mesenteric vein gas (red arrow) and dilated colon (blue arrow). (B) Gray-scale US image showed scattered hyperechoic spots with “comet tails” in the hepatic periphery and a large volume of strong-echoic gas embolus in the right anterior branch of the portal vein (arrow). (C) Color Doppler US showed an artifact in the portal vein where gas embolus partially blocked the blood flow. (L, liver; ST, stomach; CO, colon; SI, small intestine)

## Case presentation

**Fig. 2:**
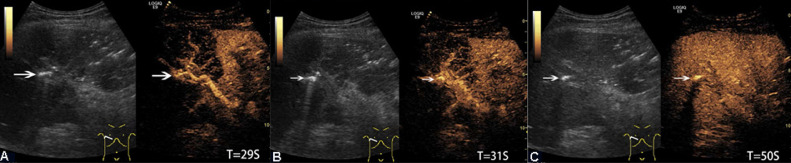
Representative images of PVG by CEUS. The right anterior hepatic lobe showed hypo-enhancement during the late arterial phase (A) and early portal phase (B). (C) During the mid-portal phase, the right anterior hepatic lobe showed iso-enhancement. Gas embolus in the right anterior branch of the portal vein remained strongly echoic during the whole CEUS process (arrow). (T, Time; S, Second)

A 64-year-old man presented to the ER with a 10-hour history of abdominal pain and fever, accompanied by diarrhea and vomiting. The patient was conscious, with a pulse of 110 beats/min and blood pressure of 82/58 mm Hg. Physical examination revealed tenderness of the right lower abdomen, accompanied by hyperactive bowel sounds. The patient had no underlying liver disease. Laboratory examination on admission showed an elevated neutrophil/ leucocyte count of 94.3%, an elevated high-sensitivity C-reactive protein level of 203.1 mg/L, and a procalcitonin level of > 100 ng/mL. Bacterial and fungal blood cultures were negative. An abdominal CT scan was performed in the ER, revealing the presence of gas in both the hepatic portal vein and mesenteric vein, as well as the colonic wall, with associated colon dilation (Fig. 1A). Bedside US examination was performed 6 hours later with a LOGIQ E9 ultrasound system (General Electric (GE) Healthcare, Wauwatosa, WI, USA) and a C1-5-D (2.78 MHz-3.7 MHz) low frequency convex abdominal transducer to closely observe any changes. Grayscale US showed abundant linear hyperechoic gas emboli collected in the portal vein and its small peripheral branches. There was also a large volume of strongly-echoic gas embolus on the right anterior branch of the portal vein, with posterior reverberation artifacts (Fig. 1B). The structure of the major portal vein in the left hepatic lobe was obscured by air-induced artifacts. A hyperechogenic gas embolus in the right anterior branch of the hepatic portal vein was indicated by turbulent flow on color Doppler imaging (Fig. 1C). For further diagnosis and assessment of liver blood flow in real time, the patient agreed to undergo CEUS. A 1.5 mL US contrast agent (SonoVue, Bracco, Italy) was injected through the antecubital vein followed by a 5 mL saline flush. The right anterior hepatic lobe showed hypoenhancement during the late arterial and early portal phases (Figs. 2A and B) relative to the normal left hepatic lobe and isoenhancement during the portal and late phases (Fig. 2C). The liver parenchyma with gas in the small peripheral branches also showed delayed enhancement during CEUS.

The clinical diagnosis of the patient was septic shock with possible bacterial translocation originating from the gastrointestinal tract. The patient received conservative treatment with fluid resuscitation, vasopressors and intravenous antibiotics. The levels of inflammatory markers gradually returned to normal, and repeated CT examination on day 4 showed total dissolution of the PVG. The patient's condition gradually improved after conservative treatment, and he was discharged 27 days later.

## Discussion

We reviewed the literature and summarized the studies that mainly reported on the ultrasonic features of PVG [3], [4], [5], [6], [7], [8], [9]. Since PVG has a rapidly changing imaging appearance, an imaging diagnosis method is appropriate for discovering disease changes and predicting patient prognosis [9]. In our case, US examination was performed 6 hours after CT. However, the distribution and sizes of the gas bubbles in the portal vein were not the same. A subsequent CT scan on day 4 revealed the total disappearance of the PVG. The reasons for the rapid change in the PVG may include the treatment of the gastrointestinal infection and the acceleration of gas dissolution by the abundant blood supply of the liver.

In some studies, grayscale US showed a centrifugal distribution of PVG in the intrahepatic lobe related to the direction and velocity of portal venous blood flow [3,5]. Other researchers reported that the shear rate of the gas bubbles, which is caused by the centrifugal direction of portal venous blood flow, resulted in gas accumulation in the liver periphery [3,4,6]. In other studies, the Doppler US features were caused by an artifact, the acoustic reflection of intravascular moving gas bubbles [3,4]. Owing to the strongly echogenic gas emboli in the right anterior trunk and small peripheral branches of the portal vein in our case, gas bubbles slowed down the blood flow, resulting in hypoenhancement of the anterior right hepatic lobe during the early portal phase of CEUS. The regional arterial hypoenhancement may have been caused by localized vasoconstriction, which could be a compensatory response to the patient's hypotension or a local hemodynamic countereffect of the gas embolism or sepsis.

In clinical practice, CEUS makes it easy to distinguish intrahepatic PVG from bile duct gas (also called pneumobilia). As in this case, PVG tends to accumulate in the periphery of the liver [3,7], leading to hypoenhancement (or delayed enhancement) of the affected liver regions during the early portal phase of CEUS. However, gas in bile ducts has a more central location than PVG, and pneumobilia does not change liver blood perfusion. PVG might be identified in the vessel lumen as intensely echogenic foci moving within the blood [3,4,8]. In contrast, the location of pneumobilia is relatively stable.

Therefore, although CEUS is strongly operator-dependent and limited by gas-induced artifacts, its field of view and low penetrability, when compared with CT and/or CECT, its advantages of high flexibility, high temporal resolution, low cost and no radiation, render it as the favorable imaging modality for the rapid bedside evaluation and monitoring of PVG in the ER.

## Patient consent

Written informed consent was obtained from the patient.
